# Socio‐economic predictors of time to care home admission in people living with dementia in Wales: A routine data linkage study

**DOI:** 10.1002/gps.5446

**Published:** 2020-10-19

**Authors:** Clarissa Giebel, Joe Hollinghurst, Ashley Akbari, Christian Schnier, Tim Wilkinson, Laura North, Mark Gabbay, Sarah Rodgers

**Affiliations:** ^1^ Institute of Population Health Sciences University of Liverpool Liverpool UK; ^2^ NIHR ARC NWC Liverpool UK; ^3^ Health Data Research UK (HDR‐UK) Data Science Building Swansea University Swansea UK; ^4^ Administrative Data Research Wales Swansea University Swansea UK; ^5^ Dementia Platform London UK; ^6^ Usher Institute University of Edinburgh Edinburgh UK; ^7^ Centre for Clinical Brain Sciences University of Edinburgh Edinburgh UK

**Keywords:** care homes, dementia, health inequalities, routine data, socio‐economic status

## Abstract

**Objectives:**

Limited research has shown that people with dementia (PwD) from lower socio‐economic backgrounds can face difficulties in accessing the right care at the right time. This study examined whether socio‐economic status (SES) and rural versus urban living location are associated with the time between diagnosis and care home admission in PwD living in Wales, UK.

**Methods/Design:**

This study linked routine health data and an e‐cohort of PwD who have been admitted into a care home between 2000 and 2018 living in Wales. Survival analysis explored the effects of SES, living location, living situation, and frailty on the time between diagnosis and care home admission.

**Results:**

In 34,514 PwD, the average time between diagnosis and care home admission was 1.5 (±1.4) years. Cox regression analysis showed that increased age, living alone, frailty, and living in less disadvantaged neighbourhoods were associated with faster rate to care home admission. Living in rural regions predicted a slower rate until care home admission.

**Conclusions:**

This is one of the first studies to show a link between socio‐economic factors on time to care home admission in dementia. Future research needs to address variations in care needs between PwD from different socio‐economic and geographical backgrounds.

## 1 INTRODUCTION

Dementia affects approximately 50 million people worldwide,^1^ and 850,000 in the United Kingdom alone.^2^ In Wales specifically, the total cost of dementia in 2013 was estimated to have been £1.4 billion per year, of which 46% was accounted for by unpaid care.^2^ Besides informal care provided by family and friends, one of the biggest cost factors in dementia is care home residency, which is 1.8 times more expensive than home care.^3^

**Key points**

- Little evidence to date on the effects of socio‐economic background on time to care home admission, with existing evidence based on small samples
- Using population scale (Wales) linked routine electronic health record data, we could investigate an under‐researched area
- People with dementia from low socio‐economic backgrounds were found to have slower rates of care home admission in Wales over 1 year
- Living in a rural as opposed to urban environment was also found to result in slower rates of care home admission

Entering a care home can be a stressful experience, by leaving the familiar home environment and getting adjusted to a new setting. Moreover, it is one of the most cost intensive element of the dementia care pathway, often resulting in people having to sell their homes to be able to afford institutional long‐term care.^4^ Therefore, people with dementia (PwD) are encouraged to stay in their own home for as long as possible. However, at some point, the care needs of a person cannot be supported sufficiently within the community any longer, for example, due to increased support needs with daily activities,^5^ so that entering a care home is the best solution to care for PwD appropriately. Other people might feel isolated at home however by living alone, and the family carer might live far away, so that a faster care home entry might be more desirable and suitable to their lifestyle. However, decisions to enter a care home depend on a variety of circumstances, including unpaid carers' and PwD's wishes as well as which country people reside in.^6^ Several studies have explored the predictors of accessing a care home in dementia, and found that difficulties with daily activities, behavioural problems, cognitive deficits, depression, reduced carer quality of life, and carer burden all predict care home admission, whereas problems with daily activities was often the most significant predictor.^7^, ^8^, ^9^, ^10^, ^11^

Considering the large costs associated with staying in a care home,^3^ accessing a care home might be difficult for people from more disadvantaged backgrounds. For Wales specifically, people with savings or financial assets worth £50k or more have to fully fund their care home stay,^12^ which can be a barrier. Across the 22 local authorities in Wales, care home provision varies with many providers owning a single care home, whilst other providers own multiple care homes and others are run by local authorities.^13^ Limited previous research indicates that people from more disadvantaged backgrounds experience health inequalities in accessing dementia care,^14^, ^15^ with a recent data linkage study reporting that PwD in general are more likely to live in a deprived area^16^ and developing dementia was higher for those people living in the most deprived areas.^17^ Van de Vorst and colleagues^15^ for example reported socio‐economic disparities in mortality after a dementia diagnosis, with people from a low socio‐economic status (SES) having higher risks of mortality. Moreover, Cooper and colleagues^14^ showed that PwD from more affluent backgrounds were 25% more likely to access anti‐dementia medication than those from more disadvantaged backgrounds. Specifically, to date, little research has looked into socio‐economic factors and individual background characteristics that might predict care home admission in dementia. It appears that only one study^10^ has found that being from a White ethnic background (and living alone) predicted increased likelihood of care home admission for people with Alzheimer's disease (AD). In their analysis of 3000+ PwD, Knapp et al.^10^ only included people with AD however and those living in one urban area, therefore limiting the generalisability of the findings. Thus, their findings provide no insight into the time to care home admission, but instead the general likelihood of admission. Yaffe and colleagues^18^ (2002) explored care home placement in PwD utilising medicare data from the United States, reporting placement likelihoods of 22%, 40%, and 52% in Year 1, 2, and 3 since diagnosis. Whilst Yaffe et al.^18^ (2002) also explored the factors contributing to placement, no focus was placed on socio‐economic background. Therefore, there is a gap in the evidence base on how SES and rural living location contribute to care home placement in dementia over a substantial period of time, both globally but also specifically in Wales. Moreover, this leaves out many other important factors of someone's socio‐economic background, such as education, income, and the level of deprivation of the neighbourhood they live in, clearly highlighting an important gap in the evidence base. The latter can be measured by the Index of Multiple Deprivation (IMD), and has been shown in other studies to be linked to differences in healthcare utilisation.^19^, ^20^

With limited evidence on socio‐economic predictors of care home admission in dementia, the aim of this study was to use linked routine electronic health record (EHR) data to explore the effects of SES and other factors on the time between dementia diagnosis and care home admission in PwD living in Wales. We hypothesised that people from disadvantaged backgrounds and those living in rural areas access a care home later. Findings from this EHR data linkage study can have implications for addressing some of the priorities of the Dementia Roadmap 2025.^21^ By understanding potential health inequalities in accessing care homes timely, we can develop possible solutions to address these barriers with findings informing policy guidance on enabling PwD from any background to access care homes, and thus the right care, more timely.

## 2 METHODS

### 2.1 Study design

We used longitudinal anonymised EHR and administrative data from the Secure Anonymised Information Linkage (SAIL) Databank (9–11) to conduct a retrospective cohort study.

### 2.2 Data sets

Our cohort was created using data held within the SAIL Databank. We used the SAIL Dementia e‐Cohort (SDEC, https://portal.dementiasplatform.uk/Home/SAILDementiaECohort)^22^ to determine who and when they received a dementia diagnosis. SDEC uses a validated algorithm to identify cases of dementia in primary care, secondary care, and mortality data. We used the Welsh Longitudinal General Practice (WLGP) data to calculate the electronic Frailty Index (eFI).^23^, ^24^ We included the Annual District Death Extract (ADDE) to determine dates of death. We used the Patient Episode Database for Wales to calculate the Charlson Index for each individual. We used the care homes data set (CARE) which flags any residence as a care home within the Welsh Demographic Service Dataset (WDSD). We used the WDSD to determine when people moved in to care homes, and to include demographic variables. Data were requested from January 2000 to December 2018.

### 2.3 Participants and sample selection

**FIGURE 1:**
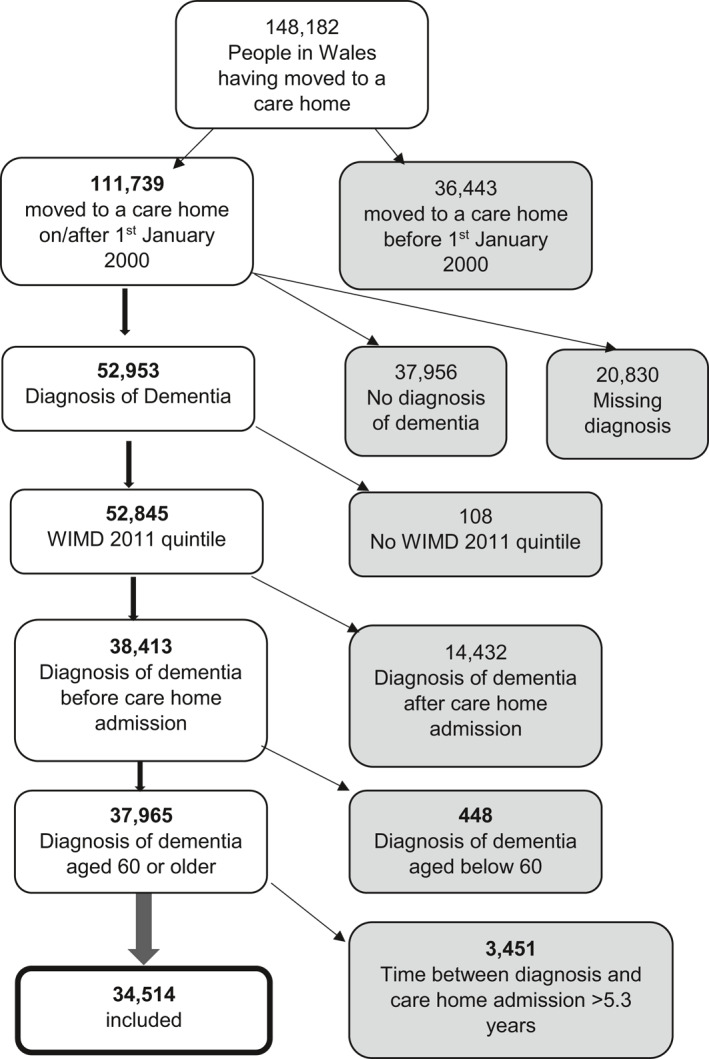
Flow diagram of sample selection

Data from people with any diagnosis of dementia having been admitted into a care home in Wales were included. Figure 1 outlines the flow of participant selection in detail, with a final sample of 34,514 included in this analysis.

### 2.4 Variables

Demographic characteristics of age and gender were obtained from the WDSD. Date of diagnosis was measured as the first clinical record of dementia in the SDEC data set, derived from the SAIL primary and secondary care data, from which we also collected data on the dementia subtype (AD dementia, vascular dementia, frontotemporal dementias, and Lewy body dementia). Each person could have more than one subtype diagnosis, so that no one diagnosis is mutually exclusive. Data on mortality was also obtained from the SDEC, derived from the SAIL ADDE mortality data.

Date of care home admission was generated by combining the WDSD with the CARE data set to create an individual‐level admission date. Living situation at time of diagnosis and living situation at time of care home admission was recorded as ‘living alone’ if the PwD had lived alone in the address preceding the diagnosis/care home admission.

Frailty was measured using the eFI, which inquires 36 deficits to identify older adults with no (fit), mild, moderate, or severe frailty at the time of care home admission, with data obtained from the WLGP.

Comorbidities were measured using the Charlson Comorbidity Index,^25^ which collects information on 22 comorbidities at the time of care home admission. A score of ‘−1’ indicates diabetes with long‐term effects, ‘0’ indicates no chronic conditions, and each comorbidity receives a score that is added up. For the purpose of this analysis, we have categorised the Charlson Index into ‘−1’ and ‘0’, 1–10, and >10.

Socio‐economic background was measured using the Welsh Index of Multiple Deprivation (WIMD) quintiles version WIMD 2011 of the last residence prior to CH admission, with ‘1’ indicating the least disadvantaged neighbourhoods, and ‘5’ the most disadvantaged neighbourhoods. The WIMD measures income, employment, health, education, access to services, housing, community safety, and physical environment in declining levels of importance to produce overall rankings of neighbourhood deprivation, and has been utilised in previous explorations of how SES is linked to health outcomes.^26^, ^27^ Rural and urban location of the living situation a day prior to care home admission was derived from the Office of National Statistics rural urban classification and linked to the Lower layer Super Output Area (LSOA) of residence version LSOA 2001, with areas of 10k population size or more classed as ‘urban’.

### 2.5 Data analysis

Data were analysed using SPSS 25, with significance level set at *p* < 0.05. Demographic variables and measures were analysed using frequency analysis. ANOVA with Bonferroni correction was employed to measure variations in the time between dementia diagnosis and care home admission amongst WIMD quintiles. An independent *t*‐test was performed to assess variations in the time between diagnosis and care home admission between those living in rural and urban locations.

Survival analysis included the Kaplan–Meier method to explore the variation of the effects of individual WIMD quintiles and rural versus urban living location on the time to care home admission. Cox regression analysis was employed to assess the effects of living situation prior to CH admission, WIMD 2011 quintile, rural and urban location, eFI, and age on the event of being admitted into a care home within 1 year since a dementia diagnosis was made.

To explore whether IMD quintile and geographical living location were associated with the time to mortality after care home admission, ANOVA with Bonferroni correction and independent *t*‐tests, respectively, were used, with time to mortality as outcome variable.

## 3 RESULTS

### 3.1 Participant characteristics

Table 1 outlines the demographic characteristics of the sample. A total of 34,514 people living with dementia in Wales were included in this analysis. PwD were on average aged 84 (±7 SD) years old, mostly female (68.3%), and lived with others prior to care home admission (82.5%). The majority of PwD had a diagnosis of AD dementia (45.0%), followed by vascular dementia (36.6%). Most PwD lived in rural environments (69.2%). Most PwD had some level of frailty at the time of diagnosis, with most people being mildly frail (32.0%). PwD took 1.5 (±1.4 SD) years from diagnosis to entering a care home, with a median of 362 days (range 0–1935). Of the sample, 50.3% were admitted into a care home within 1 year since their diagnosis (*n* = 17,355), and 69.8% were admitted within 2 years since their diagnosis (*n* = 24,089).

**TABLE 1:** Demographic characteristics

	Total sample (*n* = 34,514)
*N* (%)	
Gender	
Female	23,586 (68.3%)
Male	10,928 (31.7%)
Living situation prior to care home admission	
Lived alone	5896 (17.5%)
Lived with others	27,891 (82.5%)
Dementia subtype	
Alzheimer's disease	15,534 (45.0%)
Vascular dementia	12,633 (36.6%)
Frontotemporal dementia	270 (0.8%)
Dementia with Lewy bodies	487 (1.4%)
WIMD 2011 quintiles	
1	6436 (18.6%)
2	7569 (21.9%)
3	8182 (23.7%)
4	6788 (19.7%)
5	5539 (16.0%)
Geographical location	
Rural	21,626 (69.2%)
Urban	9643 (30.8%)
eFI at diagnosis	
Fit	13,623 (39.5%)
mild frailty	11,030 (32.0%)
moderate frailty	7186 (20.8%)
severe frailty	2675 (7.8%)
Charlson Comorbidity Index at diagnosis	
−1 to 0 (fit)	17,599 (51.0%)
1–10 (mild comorbidities)	1890 (5.5%)
10+ (severe comorbidities)	15,025 (43.5%)
Mean (SD) [range]	
Age	84 (7) [60–110]
Time between diagnosis and care home admission in months	1.5 (1.4) [0–5.3]
Time between care home admission and death	2.3 (2.3) [0–17.5]
Charlson Comorbidity Index score at diagnosis, median	0 [−1 to 83]

Abbreviations: eFI, electronic Frailty Index; WIMD, Welsh Index of Multiple Deprivation.

### 3.2 Care home admission by SES

**FIGURE 2:**
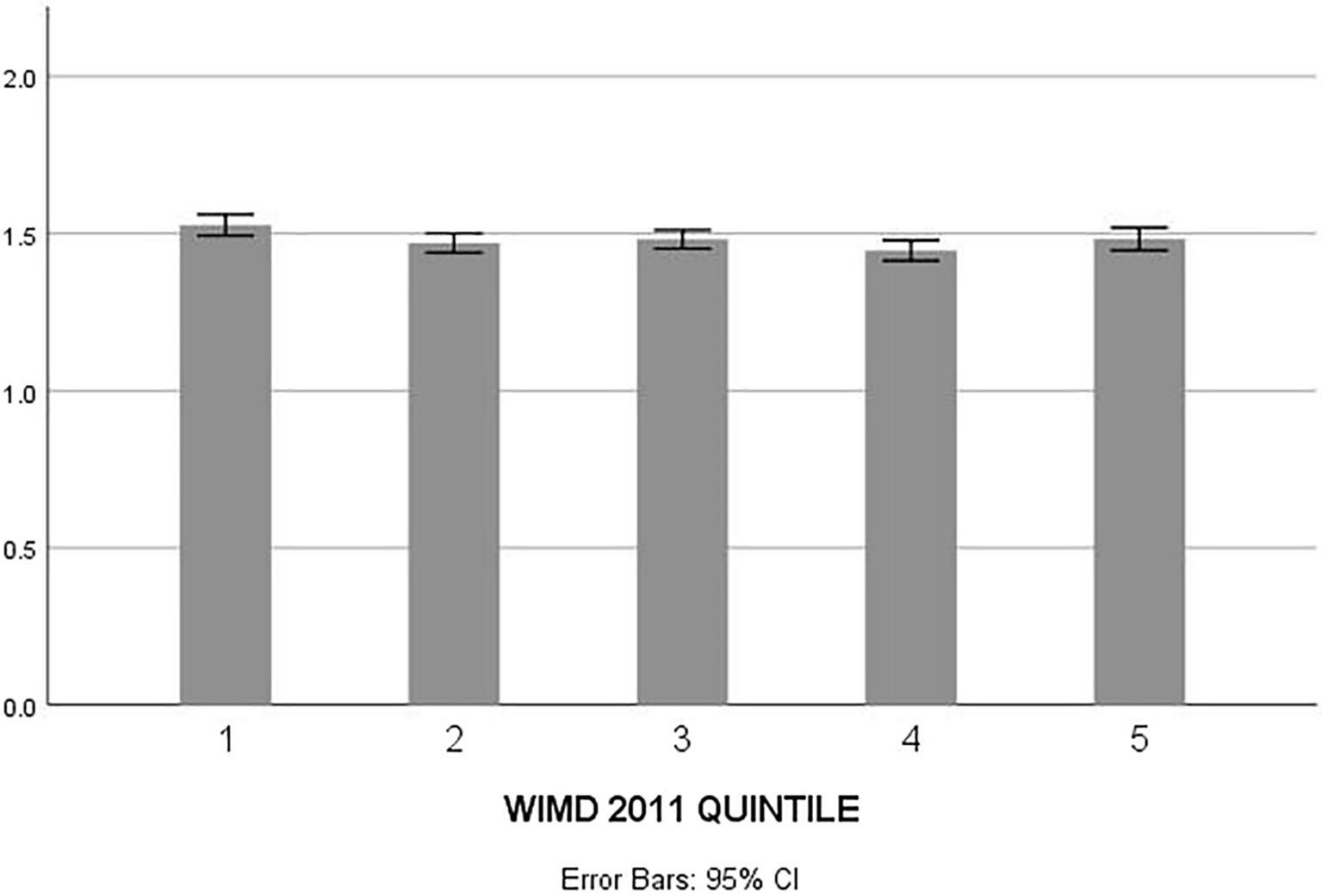
Time between diagnosis and care home admission by Welsh Index of Multiple Deprivation (WIMD) Quintile. *Y*‐axis shows the length in time in years between dementia diagnosis and care home admission

Figure 2 shows the time between diagnosis and care home admission by WIMD decile. ANOVA with Bonferroni correction showed that the time between diagnosis and care home admission significantly varied between different WIMD quintiles (F(4,34513) = 3.045, *p* = 0.016).

**FIGURE 3:**
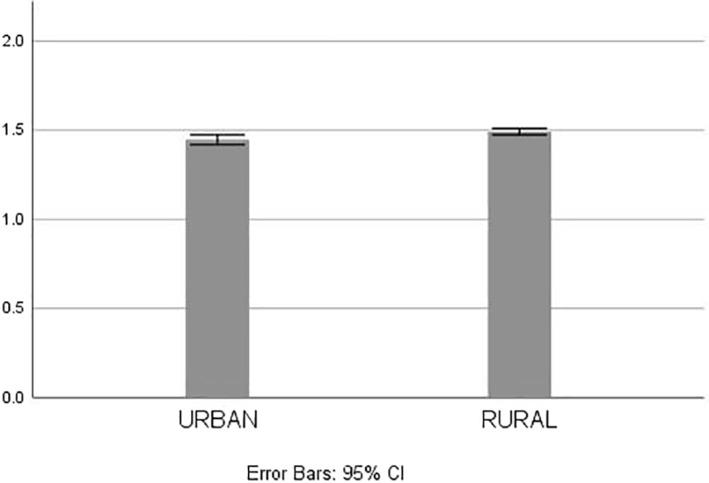
Time between diagnosis and care home admission by rural/urban indicator. *Y*‐axis shows the length in time in years between dementia diagnosis and care home admission

Figure 3 shows the time between diagnosis and care home admission in those living in rural and those living in urban locations. An Independent *t*‐test showed that the time between diagnosis and care home admission significantly varied between those living in rural versus those living in urban locations (t(31267) = 2.678, *p* = 0.007; mean difference = 0.0452; 95% confidence interval: 0.0121–0.0783), with those living in rural environments remaining slightly longer in the community before entering a care home (mean: 1.49 [±1.38] years) than those living in urban environments (mean: 1.45 [±1.38] years).

### 3.3 Variations in predictors of care home admission by SES

**FIGURE 4:**
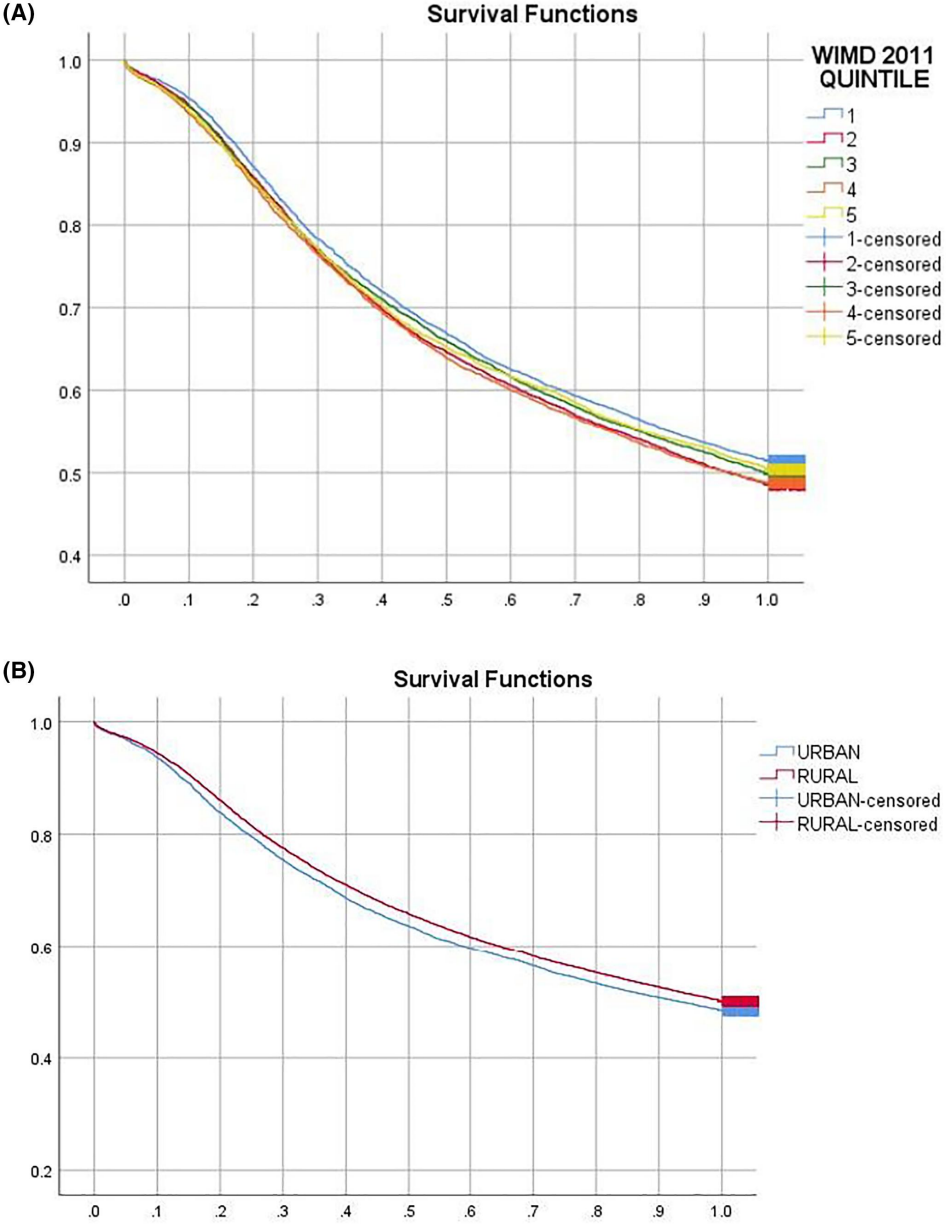
Kaplan–Meier survival curves for time until care home admission by Welsh Index of Multiple Deprivation (WIMD) Quintiles (A) and rural versus urban living location (B). *X*‐axis shows the length in time in years between dementia diagnosis and care home admission. *Y*‐axis shows the cumulative survival rate in the first 12 months since diagnosis

Kaplan–Meier survival curves showed that PwD from the most disadvantaged background had a longer duration from dementia diagnosis to care home admission compared to those living in the most advantaged neighbourhoods. PwD from rural backgrounds were delayed in entering a care home compared to those living in urban environments (see Figure 4).

Cox regression analysis showed that increased age at care home admission (hazard hatio [HR] = 1.012 [1.010, 1.015], *p* = 0.000), living alone (HR = 1.227 [1.179–1.277], *p* = 0.000), being more frail (HR_mild vs fit_ = 1.058 [1.018–1.099], *p* = 0.004; HR_moderate vs fit_ = 1.192 [1.142–1.244], *p* = 0.000; HR_severe vs fit_ = 1.378 [1.300–1.461], *p* = 0.000), and living in less disadvantaged neighbourhoods (Quintile 1) compared to the most deprived quintile (5) (HR_Q2_ = 1.070 [1.016–1.126], *p* = 0.010; HR_Q4_ = 1.056 [1.002–1.113], *p* = 0.040) predicted faster/higher rates of admission to a care home. Living in rural as opposed to urban locations prior to care home admission (HR = 0.943 [0.911–0.977], *p* = 0.001) predicted delayed/lower rates of admission into a care home.

### 3.4 Association between SESs and living location on mortality rate

ANOVA with Bonferroni correction showed no significant variations in time to death since care home admission between PwD from different IMD quintiles (F(4,28892) = 1.439, *p* = 0.218). Independent samples *t*‐test showed no significant variations in time to death since care home admission between rural and urban living location (t(26524) = 1.501, *p* = 0.133).

## 4 DISCUSSION

This is the first study using linked routine EHR data to explore the association between SES and geographical living location on time to care home admission in people living with dementia, for a whole nation. Confirming our hypothesis, PwD from more disadvantaged backgrounds and those living in more rural regions experienced a longer time between the point of dementia diagnosis and care home admission, as measured within the first 12 months since diagnosis.

Living in more disadvantaged neighbourhoods was significantly associated with slower time to care home admission in PwD across Wales. It is important to highlight that in the present study, neighbourhood deprivation was used as a proxy for individual‐level deprivation. This may result in PwD being potentially misclassified, as they may live in a more disadvantaged neighbourhood, but actually have higher levels of income and education than the average residents of this neighbourhood. However, using deprivation indices is common in health research to allow for larger, more representative, data sources, and the WIMD in particular has been used in previous explorations of how SES is linked to health outcomes or access to care.^26^, ^27^ In addition, entering a care home does not solely depend on the socio‐economic background of the PwD, but also on their family members (spouses, adult children), and their income. If the PwD cannot afford a care home place, then the family will have to contribute towards this. In the SAIL database however, no data have been collected on the socio‐economic background of family carers, which will be important to look at in future research.

Whilst there is a small, yet burgeoning, evidence base showing the effects of SES on dementia care,^28^ it appears that only very few studies have explored some social factors and their effects on care home admission in PwD.^10^ Limited research has specifically explored the predictors of time to care home admission, yet not focused on SES and was limited by its small sample of people with AD and Lewy Body dementia;^29^ Yaffe et al., 2002.^18^ The timeliness of the initial dementia diagnosis can be a limitation in the present study, as it is well known that there are severe delays in getting a diagnosis in the first place.^30^ This means that some people might receive a diagnosis whilst already being in the more advanced stages of the condition, whilst others might have received a diagnosis more quickly, which will inevitably have an implication on the time to care home placement. Unfortunately, no data were available on first symptom recognition, as this data linkage used routinely collected data. Further research needs to link up routine data with additional primary data collection on first symptom recognition, or link up with data on the severity of dementia. Nevertheless, the present findings not only contribute novel evidence to a growing research field, but can also have important implications for policy guidance on care homes and their potential (self‐) funding.

There are likely multiple underpinning reasons as to why people from lower SE backgrounds access care home at a later stage. One reason might be the high costs. Specifically, in Wales, people have to fully fund their care home stay if they have savings or similar financial values worth £50k or higher,^12^ so that people may be more inclined to prolong care at home. It is important to note however that this threshold has changed over the 20‐year period of this investigation, as the threshold has only recently been changed from £40k to £50k. This may result in increased care needs though prior to care home admission, something that this routine data linkage study was unable to assess. By using time to death as a proxy measure of severity in this study, we found no significant variations between time to death after care home admission amongst different levels of deprivation. It is also important to highlight that this analysis was based on data from almost 2 decades, in which care commissioning for example might have changed in individual local authority areas. However, looking at data spanning this time period provides a strong data set and powerful sample to explore this under‐researched topic to date. Future research is recommended to explore potential variations in care needs between PwD from more and less disadvantaged backgrounds at the point of care home entry.

Geographical location was also found to be linked to the time to admission with PwD living in rural areas entering a care home at a slower rate. Slower entry rate is not necessarily negative, as people may be cared for better at home. Moreover, with care homes being further away when living in rural regions, PwD may not wish to move into a care home that is out of their neighbourhood, or, their family does not wish their relative with dementia to be living in a care home far away. The relatively small effect size in the regression model is worth noting, suggesting that whilst living location is significantly associated with time to care home admission, other factors have a larger effect, such as frailty. Previous literature on the impact of rurality on dementia care has shown that living in more rural regions is often linked to unmet needs.^31^, ^32^, ^33^ Similar to SES, this data set does not inform about the level of care needs, and the specific underpinning reasons as to why rurality affects time to care home admission. Whilst frailty as a measure of the physical condition of the PwD is included in this analysis, there are many other care needs in dementia which have been found to contribute to care home admission, including everyday functioning, behavioural problems, and cognitive deficits.^34^ Qualitative investigations may therefore provide crucial insights.

In addition to socio‐economic and geographical factors, living alone also contributed to faster rates of care home admission. This corroborates previous literature,^8^, ^10^ and suggests unmet care needs by living alone. Unpaid carers provide the greatest share of care,^1^, ^2^ which equates to an estimated £13.9 billion each year in the UK. However, once family carers experience high levels of burden,^35^ their relative with dementia is often admitted into a care home,^36^ suggesting that family members should be supported to cope and have a good quality of life to enable their relative to stay well in the community for longer. For those PwD who live alone, better access to post‐diagnostic community support services needs to be put in place so that PwD can access the support they need.

### 4.1 Limitations

This study was based on routinely collected EHR data linked with a specifically designed care home data. The care home data (CARE) was created using data of current care homes in the Care Inspectorate Wales database in 2018. By using routine data, no data are included on severity of the dementia. In addition, the date of diagnosis is based on the first clinical record of dementia. However, people may have been diagnosed before this date, and people may have been delayed in going to their doctor to get a diagnosis, possibly due to their SES. The sensitivity of using UK routinely collected primary care, hospital admissions and mortality data in combination to identify PwD is not known.^37^, ^38^ It is likely that a proportion of ‘true’ dementia cases would have been misclassified as non‐cases. Under‐recording of dementia in EHR data may itself be related to SES. Data linkage also resulted in 20,000+ missing cases on dementia diagnosis in this study, which however did not affect the power of the sample, as we were still able to include 34,514 PwD. Considering the analysis, we acknowledge the simplicity of the Kaplan–Meier analysis and the limitation of only including a single explanatory variable, it was our aim to give a representation of the differences between levels of SES over time. The Cox regression models extended this analysis to incorporate additional variables. Lastly, we used the WIMD as a neighbourhood deprivation index, which does not provide individual‐level data on for example income and education. The mechanisms between neighbourhood and individual level of deprivation might vary however, which needs to be considered as a limitation. It is important to highlight though that many studies employ a deprivation index as opposed to individual‐level data, so that our study is not an exception.

## 5 CONCLUSIONS AND IMPLICATIONS

This is the first study based on population scale (Wales) linked routine HER data to show how SES and geographical living location are associated with time to care home admission in PwD. Future research needs to explore the underlying reasons for these relationships, and variations in care needs at care home admission. These findings clearly address the Dementia Roadmap 2025,^21^ and provide novel insights of existing health inequalities in dementia care, by addressing one of the five essential conditions for more equal health for all as outlined in the recently published WHO European Health Equity Status report.^39^

## CONFLICT OF INTEREST

None.

## Data Availability

The data that support the findings of this study are available from the corresponding author upon reasonable request.
